# Historical Benchmarks for Quality Tolerance Limits Parameters in Clinical Trials

**DOI:** 10.1007/s43441-021-00335-3

**Published:** 2021-08-27

**Authors:** Marcin Makowski, Ruma Bhagat, Soazig Chevalier, Steven A. Gilbert, Dagmar R. Görtz, Marta Kozińska, Patrick Nadolny, Melissa Suprin, Sabine Turri

**Affiliations:** 1 Global Clinical & Data Operations, GlaxoSmithKline GmbH & Co. KG, Prinzregentenpl. 9, 81675 Munich, Germany; 2 Product Development Quality, Roche, 1 DNA way, South San Francisco, CA 94404 USA; 3 Clinical Sciences and Operations, Sanofi, 1 Avenye Pierre Brossolette, 91380 Chilly-Mazarin, France; 4 Statistical Research & Innovation, Pfizer Inc., 300 Technology Square, Third Floor, Cambridge, MA 02139 USA; 5 BioResearch Quality & Compliance, Janssen-Cilag GmbH, Johnson & Johnson Platz 1, 41470 Neuss, Germany; 6 Centralized Monitoring, AstraZeneca, Postepu 14, 02-390 Warsaw, Poland; 7 Clinical Data Management, Sanofi, 1 Avenue Pierre Brossolette, 91380 Chilly-Mazarin, France; 8 Clinical Data Management and Programming, Allergan, 2525 Dupont Drive, Irvine, CA 92612 USA; 9 Clinical Development Quality Center of Excellence, Pfizer, Inc, Eastern Point Road, Groton, CT 06340 USA; 10 Global Development Operations, Trial Management, Novartis Pharma S.A.S., 92506 Pueil-Malmaison Cedex, France

**Keywords:** Quality tolerance limits, QTL, Historical data, Thresholds, Clinical trials

## Abstract

**Background:**

In 2016, the International Council for Harmonisation of Technical Requirements for Pharmaceuticals for Human Use updated its efficacy guideline for good clinical practice and introduced quality tolerance limits (QTLs) as a quality control in clinical trials. Previously, TransCelerate proposed a framework for QTL implementation and parameters. Historical data can be important in helping to determine QTL thresholds in new clinical trials.

**Methods:**

This article presents results of historical data analyses for the previously proposed parameters based on data from 294 clinical trials from seven TransCelerate member companies. The differences across therapeutic areas were assessed by comparing Alzheimer’s disease (AD) and oncology trials using a separate dataset provided by Medidata.

**Results:**

TransCelerate member companies provided historical data on 11 QTL parameters with data sufficient for analysis for parameters. The distribution of values was similar for most parameters with a relatively small number of outlying trials with high parameter values. Medidata provided values for three parameters in a total of 45 AD and oncology trials with no obvious differences between the therapeutic areas.

**Conclusion:**

Historical parameter values can provide helpful benchmark information for quality control activities in future trials.

## Introduction

In 2016, International Council for Harmonisation of Technical Requirements for Pharmaceuticals for Human Use (ICH) E6 introduced quality tolerance limits (QTLs) as a quality control in clinical trials. Since then, the industry has been learning about implementation. In support of this learning, TransCelerate prepared this manuscript to offer a frame of reference for future clinical trials.

QTLs set limits for detection of systematic issues impacting on participants’ safety or the reliability of trial results. Several recent papers fill in some of the missing details about implementing QTLs, including their relationship to risk-based monitoring [1] and a framework for implementation [2]. Using a risk-based approach, sponsors typically define which of their clinical trials will benefit from QTLs as a risk control. Each clinical trial then sets QTLs by defining the parameters to be observed, the units of measure, the data source(s), and their initial limit(s). TransCelerate’s QTL Framework also contains examples of parameters [2].

While many of the parameters are familiar to those involved in clinical trials, using them to set quality limits and to guide quality decisions is still new. Implementation of QTLs brings new challenges in data specifications, collection, and analysis. Defining parameters and setting limits for a trial can be challenging as those need to be predefined (i.e., set before a trial starts and data are accumulated). Historical data, clinical and statistical expertise, and an understanding of the protocol can be helpful in considering how to set the thresholds for QTLs in a given trial. This manuscript was written to expand the available references that sponsors can consult when setting QTLs by providing historical benchmarks of parameter values.

In 2020, TransCelerate published an expanded and updated framework for QTL implementation (available at www.transceleratebiopharma.com and published as a peer-reviewed article [2]). The QTL parameters proposed by Bhagat et al. were used as a basis for this article [2]. TransCelerate collected and aggregated historical data provided by a subset of TransCelerate member companies and separately by Medidata. As the list of parameters used is predominantly applicable for medium and large trials, therefore, this exercise focused on trials recruiting more than 50 trial participants. At the time this study was designed, the volume and characteristics of the data to be received was unknown; thus, no statistical hypothesis testing was planned.

Importantly, the objective of this article is not to provide recommendations on what parameter values should be applied as QTLs for future trials. These need to be independently determined by every sponsor. Instead, we are providing historical benchmark information on five different parameter values that can be especially useful in conjunction with a sponsor’s internal data and research when a sponsor utilizes the concept of ‘expectation’ as introduced in a 2017 TransCelerate publication on QTLs [3].

## Methods

### Historical Data Collection from TransCelerate Member Companies

Companies were asked to complete a data collection tool without using trial identifiers (e.g., trial name) so that data could be aggregated blindly. The data contribution was voluntary. Key trial characteristics captured for each trial were therapeutic area, phase, number of participants (categorized as less than 20, 21–50, 51–200, 201–1000, or more than 1000), route of drug administration, trial design, and when the trial was completed (within last five years vs. more than five years ago). Trial characteristics that were not available were left blank. Along with trial characteristics, this tool collected QTL parameters measured in each trial and their units and values. Each record (row) within the tool represented one parameter per trial. If a trial was assessed for multiple parameters, multiple lines were completed with the required information. As this was historical data collection, the parameters could be assessed after trial completion and were not used for monitoring the trial actively. Data collection tools completed by each responding member company were shared with the TransCelerate Project Manager, an independent consultant retained by TransCelerate to oversee the QTL initiative. For all planned aggregations, dataset balancing exercise was performed to prevent results being disproportionally driven by one member company. For this purpose, the Project Manager (1) calculated the percentage of data from each member company; (2) defined the number of study records that need to be removed from the sets coming from top-contributing member companies to achieve a balanced dataset; and (3) assigned a random number to every record in a company dataset and removed records starting with the smallest random number and moving higher, until the number of records defined in step 2 had been removed. The activity was performed for each reported parameter independently. The Project Manager then aggregated and anonymized the data based on the authors’ instructions prior to being shared with the QTL team for analysis and interpretation. The aggregated results before and after the balancing exercise were consistent.

Aggregations were conducted without formal statistical analyses or hypotheses testing. The majority of responding companies provided data on parameters as a percentage of trial participants and the exact number of trial participants was not collected; therefore, the results were presented as a mean of percentages.

### External Partner Data Collection

Clinical trials targeting specific indications were selected from the Medidata Enterprise Data Store (MEDS), comprised of 22,000 + historical clinical trials, for de-identified aggregate analyses. In order to identify differences in parameter values between different therapeutic areas, Alzheimer’s disease (AD) and oncology trials were compared. Selection of these indications was based on the fact that AD and oncology trials differ significantly and on the availability of the data. A Medidata statistician performed the analysis based on authors’ instructions. The information on protocol deviations (PDs) was retrieved from the disposition dataset and represents the disposition-related protocol deviations.

Aggregations were conducted without formal statistical analyses or hypotheses testing.

## Results

### Historical Data Collection from TransCelerate Member Companies

Seven TransCelerate member companies provided historical data from a total of 294 trials on parameters previously proposed in the TransCelerate QTL Framework [2]. Each company provided data on 4–119 trials. The trials were predominantly late phase (72% Phase 2 or 3) and represented a wide range of therapeutic areas. The details of the trials are presented in Table 1.

**Table 1:** Characteristics of TransCelerate Member Company Trials from which QTL Parameter Data were Collected

Trial characteristics	Number (percent) of trials
*All trials*	272 (100%)
*Therapeutic area*	
Immunology	77 (28%)
Neurology	75 (28%)
Other (cardiovascular, endocrinology, oncology, psychiatry, respiratory, and other)	120 (44%)
*When completed*	
Last 5 years	179 (66%)
More than 5 years ago	43 (16%)
Missing data	50 (18%)
*Phase*	
1	58 (22%)
2	38 (14%)
3 (including IIIb)	159 (58%)
Other (2a, 2b, 4, and other)	17 (6%)
*Design*	
Parallel design	73 (27%)
Run-in design	1 (0%)
Other (cross-over design and other)	6 (2%)
Missing data	192 (71%)
*Route of administration*	
Oral	67 (25%)
Subcutaneous	59 (22%)
Intravenous	26 (10%)
Other (intramuscular, transdermal, and other/unknown/mixed)	19 (7%)
Missing data	103 (38%)
*Number of trial participants*	
21–50	27 (10%)
51–200	66 (24%)
201–1000	134 (49%)
Other (0–20 and 1000 +)	45 (17%)

The companies provided data on 2–10 parameters. The number of companies providing data on each parameter is presented in Table 2. In all further aggregations at data contributed by at least five member companies are presented. This criterion was met for five parameters.

**Table 2:** Number of TransCelerate Member Companies Providing Data by Parameter

Parameter	Number of companies providing data
Percentage of trial participants randomized who do not meet inclusion/exclusion criteria	7
Percentage of trial participants with premature discontinuation of investigational drug	6
Percentage of trial participants with withdrawal of informed consent	5
Percentage of lost to follow-up trial participants	6
Percentage of trial participants for whom trial endpoint data were not collected	5
Percentage of trial participants with important protocol deviations other than eligibility	3
Percentage of trial participants on rescue medication	1
Percentage of trial participants who are non-compliant with investigational drug administration as defined in the protocol	1
Percentage of trial participants censored for primary objective analysis	2
Percentage of randomized trial subjects who were incorrectly stratified	2

These aggregated data for parameters and trial subsets are presented in Table 3. The lowest mean value for the whole dataset was collected for the percentage of lost to follow-up trial participants and highest for the percentage of trial participants with premature discontinuation of investigational drug. The possibility of comparisons between various trial characteristics was limited by the availability of sufficient data. Nevertheless, some association between the size of the trial and the percentage of trial participants with premature discontinuation of investigational drug was noted with larger trials having higher rates of discontinuation. Otherwise, no meaningful differences in parameter values were linked with collected trial characteristics.

**Table 3:** Results of Analyses of TransCelerate Member Company Historical Data

Trial characteristics	Parameter														
	Percentage of lost to follow-up trial participants			Percentage of trial participants for whom study endpoint data were not collected			Percentage of trial participants randomized who do not meet inclusion/exclusion criteria			Percentage of trial participants with premature discontinuation of investigational drug			Percentage of trial participants with withdrawal of informed consent		
	Mean (± SD)	Median	*N*	Mean (± SD)	Median	*N*	Mean (± SD)	Median	*N*	Mean (± SD)	Median	*N*	Mean (± SD)	Median	*N*
All trials	1.78% (± 2.74%)	0.80%	124	12.58% (± 13.11%)	7.90%	23	6.70% (± 10.49%)	3.21%	141	16.03% (± 16.64%)	10.70%	182	3.95% (± 3.67%)	2.90%	50
Completed															
≤ 5 years ago	1.99% (± 3.00%)	0.81%	82	6.43% (± 5.90%)	5.20%	16	7.57% (± 14.02%)	3.20%	40	9.40% (± 8.40%)	6.62%	36	4.98% (± 5.56%)	3.20%	30
> 5 years ago	1.60% (± 2.02%)	0.80%	23	Not applicable^a^			4.21% (± 5.03%)	1.70%	17	Not applicable^a^			Not applicable^a^		
Phase															
III	2.66% (± 3.05%)	1.27%	86	12.05% (± 15.01%)	7.30%	20	7.87% (± 11.09%)	4.30%	96	17.22% (± 16.65%)	11.52%	119	6.01% (± 7.18%)	3.95%	46
Design															
Parallel	Not applicable^1^			10.99% (± 12.28%)	8.35%	20	3.45% (± 4.01%)	1.50%	20	Not applicable^a^			Not applicable^a^		
Other	1.54% (± 2.60%)	0.75%	12	Not applicable^a^			5.58% (± 6.10%)	3.33%	46	17.23% (± 16.75%)	10.70%	47	0.60% (± 0.96%)	0.00%	7
Route of administration															
Oral	1.45% (± 1.94%)	0.70%	36	Not applicable^a^			5.11% (± 5.48%)	2.20%	33	Not applicable^a^			Not applicable^a^		
Subcutaneous	Not applicable^1^			Not applicable^a^			3.55% (± 3.77%)	1.90%	13	Not applicable^a^			Not applicable^a^		
Therapeutic area															
Cardiovascular and neurology	2.31% (± 3.92%)	0.69%	14	Not applicable^a^			4.43% (± 4.89%)	3.20%	27	Not applicable^a^			Not applicable^a^		
Other	1.54% (± 2.13%)	0.80%	108	7.52% (± 8.52%)	5.10%	17	6.60% (± 11.10%)	3.20%	114	15.58% (± 16.78%)	10.50%	149	5.09% (± 6.57%)	3.40%	46
Trial size															
1000 +	2.71% (± 3.34%)	0.90%	16	Not applicable^a^			5.19% (± 4.26%)	4.08%	10	Not applicable^a^			Not applicable^a^		
201–1000	2.85% (± 3.22%)	1.55%	80	12.45% (± 13.13%)	8.27%	16	8.03% (± 12.06%)	5.10%	73	17.41% (± 17.61%)	11.15%	82	6.10% (± 5.38%)	4.60%	30
51–200	1.03% (± 1.39%)	0.75%	20	Not applicable^a^			4.19% (± 4.98%)	3.65%	23	11.26% (± 11.09%)	9.20%	33	Not applicable^a^		

*N* number of trials; *SD* standard deviation

^a^Less than five companies reporting data

The distribution of values was similar for most parameters with clustering at or near zero and a relatively small number of outlying trials with high parameter values. This pattern was less visible for percentage of trial participants with premature discontinuation of investigational drug.

### External Partner Data Analysis

As comparisons between different therapeutic areas and other trial characteristics were limited in the member company historical data collection, this was addressed using an alternate source of data. Medidata provided values for three parameters in 45 AD and oncology historical trials. The information on classification of all PDs was incomplete in the available datasets; therefore, the percentage of all patients with disposition-related PDs was assessed.

This analysis did not show any striking differences between the AD and oncology trials in any of the three analyzed parameters. However, there was a somewhat higher mean percentage of patients withdrawing consent in the oncology trials (Table 4). Also, trial characteristics (phase, design, and time of completion) did not appear to correlate with historical parameter values within the oncology dataset (Table 5). In general, for the three parameters aggregated, the mean parameter values were higher than medians in this dataset because of the small number of trials reporting high and outlying parameter values.

**Table 4:** Results of Historical Data Analysis by Therapeutic Area—Medidata Data Collection

Trial characteristics	Percentage of trial participants								
	Lost to follow-up			Withdrew informed consent			With protocol deviations		
	Mean (± SD)	Median (IQR)	*N*	Mean (± SD)	Median (IQR)	*N*	Mean (± SD)	Median (IQR)	*N*
All trials	1.1% ± 2.4%	0% (0%, 1.1%)	45	7.6% ± 7.6%	5.8% (2.1%, 10.3%)	45	0.9% ± 2.7%	0% (0%, 0.6%)	28
Oncology	1.2% ± 2.5%	0% (0%, 1.1%)	38	8.0% ± 8.0%	6.6% (1.3%, 10.3%)	38	1.1% ± 3.1%	0% (0%, 0.2%)	21
Alzheimer’s	0.5% ± 0.6%	0.2% (0.1%, 1.1%)	7	5.9% ± 5.5%	2.5% (2.4%, 13.0%)	7	0.6% ± 0.5%	0.4% (0.2%, 1.2%)	7

*IQR* interquartile range, *N* number of trials, *SD* standard deviation

**Table 5:** Results of Historical Data Analysis, Oncology Trials by Phase, Interventional Model, and Trial Completion Date—Medidata Data Collection

Trial characteristics	Percentage of trial participants								
	Lost to follow-up			Withdrew informed consent			With protocol deviations		
	Mean (± SD)	Median (IQR)	*N*	Mean (± SD)	Median (IQR)	*N*	Mean (± SD)	Median (IQR)	*N*
*Phase* (all)	0.8% ± 1.7%	0% (0%, 0.6%)	35	8.1% ± 8.0%	6.9% (1.3%, 10.3%)	35	1.1% ± 3.2%	0% (0%, 0.1%)	20
1	0.9% ± 1.8%	0% (0%, 1.5%)	11	10.5% ± 10.3%	8.3% (1.5%, 15.6%)	11	Not applicable^a^		
2	0.8% ± 2.2%	0% (0%, 0%)	15	7.9% ± 8.1%	8.0% (0.6%, 11.1%)	15	2.3% ± 4.6%	0% (0%, 0.9%)	9
3	0.5% ± 0.6%	0.5% (0%, 0.7%)	9	5.4% ± 2.8%	6.3% (3.7%, 7.2%)	9	0.1% ± 0.2%	0% (0%, 0.1%)	8
*Intervention model (all)*	1.2% ± 2.5%	0% (0%, 1.1%)	38	8.0% ± 8.0%	6.6% (1.3%, 10.3%)	38	1.1% ± 3.1%	0% (0%, 0.2%)	21
Parallel assignment	1.0% ± 2.2%	0.2% (0%, 0.7%)	17	5.2% ± 3.3%	5.8% (2.5%, 8.0%)	17	0.1% ± 0.3%	0% (0%, 0.1%)	12
Single group assignment	1.4% ± 2.8%	0% (0%, 1.5%)	21	10.2% ± 9.8%	10.0% (0%, 15.6%)	21	2.3% ± 4.6%	0% (0%, 1.3%)	9
*Trial completion date (all)*	1.2% ± 2.5%	0% (0%, 1.1%)	38	8.0% ± 8.0%	6.6% (1.3%, 10.3%)	38	1.1% ± 3.1%	0% (0%, 0.2%)	21
Within past 5 years	1.0% ± 2.3%	0% (0%, 0.6%)	16	6.5% ± 6.6%	5.7% (1.4%, 8.3%)	16	1.2% ± 3.8%	0% (0%, 0.1%)	8
Over 5 years ago	1.5% ± 2.9%	0% (0%, 2.0%)	22	9.6% ± 9.2%	8.1% (1.3%, 14.3%)	22	0.9% ± 2.1%	0% (0%, 0.6%)	13

*IQR* interquartile range, *N *number of trials, *SD* standard deviation

^a^Not enough data to report

## Discussion

Historical data analyses, together with medical and statistical characteristics of a trial, were previously proposed as factors to be considered in setting QTLs [3]. Many sponsors conduct historical data analyses using data from their own completed trials in order to generate benchmark data for setting QTLs in new trials. This activity may be difficult or impossible when a sponsor has a small portfolio of trials or when an organization is entering a new therapeutic area. This article shares benchmark data on parameters proposed as the basis for QTLs and summarizes the availability of public domain data that could be helpful in determining the historical values of the parameters.

For the purpose of this study, we collected historical data from several TransCelerate member companies. The first TransCelerate publication on QTLs was released in 2017 [3]. It included examples of parameters: percentage of ineligible participants, percentage of premature treatment discontinuations, and percentage of participants lost to follow-up. These are some of the same parameters for which the most responding member companies provided data for this publication. The data collection period for the article was short, and the member companies were encouraged to share data that were readily available. Therefore, the parameters most represented in the collected historical dataset may reflect the ease of data collection or frequency of use in QTL implementation projects.

The parameters proposed by Bhagat et al. [2] can be grouped into those that revolve around missing data (including those due to withdrawal of consent) and those that pertain to PDs. The amount of data published on each type of parameter differ significantly with more data available for parameters associated with missing data than for PD parameters.

Missing data from participants lost to follow-up were addressed in multiple previous publications from a variety of therapeutic areas [4–8] with rates of participants lost to follow-up consistent with the data gathered for this publication. In addition, Fewtrell (2008) and Kristman (2004) modeled loss to follow-up at similar rates to what is reported here [9, 10]. Each of these publications had a different definition of loss to follow-up and covered a variety of therapeutic areas; however, this is representative of our industry.

The value of loss to follow-up as a QTL that is measured, monitored, and managed is highlighted in a 2011 literature review [11]. The literature review concluded that loss to follow-up might change conclusions written in peer-reviewed published study results in 15% of randomized clinical trials with time to event outcomes. In addition, some trials do not adequately report loss to follow-up [11]. Crutzen et al. addressed differential attrition across treatment arms of a trial; they reviewed 100 randomized clinical trials in general and internal medicine and found a mean attrition rate of 13%, but no significant differential attrition [4].

In contrast to the parameters pertaining to missing data, the public domain information on eligibility-related and other PDs is very scarce and different from the results reported here. The clinical trials for which information was provided by TransCelerate Member Companies had a mean of 6.70% (median: 3.21%) of participants with eligibility-related PDs (Table 3). Sweetman et al. reviewed a series of major medical journal publications and reported a mean of 0.8% (range 0.16 to 9.1%) of ineligible participants [12]. However, these PDs were explicitly reported only in 12.5% of reviewed trials and incompletely reported in a further 8.8% of reviewed trials [12]. Kohara et al. examined the frequency and reasons for exclusion of participants from Per Protocol (PP) analysis sets in Pharmaceuticals and Medical Devices Agency (PMDA) new drug approval applications approved in fiscal years 2014 and 2015 [13]. The frequency of exclusions from the PP set due to not meeting eligibility criteria was very low with most trials not reporting any exclusions for this reason (median 0%) [13] [N. Kohara, personal communication]. Nevertheless, also in the Kohara study, the data availability was limited with only 102 trials out of 492 having the reasons for excluding participants from the PP efficacy analysis described in the summary of new drug application documents [13].

The disparity between our results (Table 3) and published previously results on PDs may be explained by the differences in data collection methods. The referenced articles used the public domain data that were available only for a minority of trials which Sweetman and colleagues consider suboptimal [12]. In contrast, data on eligibility-related PDs were available for the majority of analyzed trials (125 out of 226) for which TransCelerate Member companies provided data. Additionally, the difference between our results and the study by Kohara et al. may be explained by the fact that in the Kohara et al. study, only eligibility PDs resulting in exclusion from the PP analysis were analyzed. Those, depending on study statistical analysis plan, may be just a part of all eligibility PDs [13].

Also, both our study and the Sweetman et al. study [12] found significant variability in the percentage of ineligible participants with a small subset of trials having high percentages of participants with eligibility PDs. Trials in our analysis preceded the existence of industry-wide guidance on defining and reporting of PDs [14]. The sponsors did not receive any instructions for categorization of the PDs (minor or major) or inclusion/exclusion of minor PDs in the calculations of the percentages of participants with eligibility PDs. It can be speculated that the small number of trials reporting outliers for this parameter had a systematic issue with eligibility PDs. Standardizing PDs and establishing QTLs as a control may have had a beneficial effect on trial quality.

The public domain can be a rich source of historical data for QTL parameters that pertain to completeness of observations. Introduction of CONSORT diagrams in the majority of publications and disposition tables in results published in trial registries allows retrieval of information on patients withdrawing consent and lost to follow-up in the majority of historical trials [15]. On the other side of the spectrum are the parameters for which PD information in the public domain is very limited and therefore subject to selection bias. The availability of data on completeness of intervention reflected in parameters pertaining to treatment compliance and premature discontinuations is somewhere in the middle.

In addition to calculating historical benchmarks for parameters that may provide some basis for QTLs, it may be important to understand how various trial characteristics affect the parameter values. In the data collected from the TransCelerate member companies, the only notable difference within the dataset based on study characteristics was observed for the percentage trial participants with premature discontinuation of investigational drug. The mean parameter value was higher for larger trials. This finding is difficult to interpret without analyses of other trial characteristics. It can be hypothesized that larger trials usually represent later phases of development with longer treatment periods and more real-life settings making premature drug discontinuations more prevalent. Alternatively, the size of a trial may correlate with the therapeutic area, e.g., late phase oncology trials are often in the 201–1000 participants size category and are known to have high treatment discontinuation rates due to adverse events [16, 17]. The comparison of AD and oncology trials performed by Medidata did not show any striking difference between these two therapeutic areas in percentage of lost to follow-up trial participants and percentage of trial participants with protocol deviations. The slightly higher number of patients withdrawing consent in oncology trials can be purely due to chance. It may also reflect the fact that oncology patients often seek additional lines of treatment and may change care centers after progression. This, in combination with study burden and mortality, can make obtaining full follow-up on patients more difficult.

Overall, the findings in this paper did not provide a definitive answer to the question of whether trial characteristics, such as phase, therapeutic area, and trial size, have a significant impact on historical values of parameters proposed for QTLs. More studies and analyses are needed to answer the question. The data presented in this article may, however, make the planning and hypotheses formulation for such work easier.

The analyses presented in this article have some limitations. As this was the first look at historical data in the context of parameters explored by TransCelerate, conclusions were limited by the lack of hypothesis testing. The survey methodologies, the large variance of trials for which TransCelerate member companies provided data, Medidata dataset being limited to only AD and oncology trials altogether limited comparisons between different therapeutic areas, drug administration routes, and other trial characteristics. While the authors attempted to analyze trials representative of the types for which parameters proposed by Bhagat et al. were intended [2], the analyzed trials are just a fraction of the studies performed every year; therefore, selection bias cannot be excluded.

Historical parameter values presented here provide clinical trial sponsors with benchmark information that may help sponsors in setting QTLs in new clinical trials. In addition, public domain data on historical values of parameters proposed for QTLs are available, but variable in terms of exactly what is presented across trials. Introduction of CONSORT diagrams in the majority of publications and disposition tables in results published in trial registries allows retrieval of information on patients withdrawing consent and lost to follow-up in the majority of historical trials [15]. On the other side of the spectrum are the parameters for which PD information in the public domain is very limited and therefore subject to selection bias. Regulatory authorities (e.g., United States Food and Drug Administration [US FDA]) are signaling potential revisions of approaches to trial results disclosure regulations to further align reporting on this type of information [18]. Also, industry forums and consortia may develop data exchange platforms that include historical data pertaining to PDs. Regardless, the body of the historical data available to establish QTLs in new trials will continue to grow in the future.

## Conclusion

The first TransCelerate publication on QTLs included examples of parameters [2]. For this paper, we collected historical data on these parameters from clinical trials conducted by TransCelerate member companies and Alzheimer’s disease and oncology trials in a dataset provided by Medidata and shared benchmark data on parameters that may serve as the basis for QTLs. We did not provide a definitive answer to the question whether trial characteristics have a significant impact on historical values of parameters proposed for QTLs. More studies and analyses are needed to answer the question.
